# Caseous Calcification of the Mitral Valve

**DOI:** 10.5334/jbsr.3138

**Published:** 2023-05-02

**Authors:** Birgitt Janssens, Thiebault Saveyn, Benjamin Leenknegt

**Affiliations:** 1 KULeuven, BE; 2 AZ Sint-Lucas Gent, BE

**Keywords:** caseous mitral valve calcification, CMAC, computed tomography, transthoracic ultrasound

## Abstract

**Teaching Point:** Caseous calcification of the mitral annulus is a benign mass-like lesion which can mimic cardiac tumor or abscess; therefore, multimodal imaging should be considered, avoiding unnecessary interventions.

## Case History

A 79-year-old female patient with a history of cardiac hypertension presented to the emergency department with abdominal pain. On abdominal computed tomography (CT), a left-sided colitis was demonstrated. Also, an oval-shaped cardiac mass was noted.

**Figure 1:**
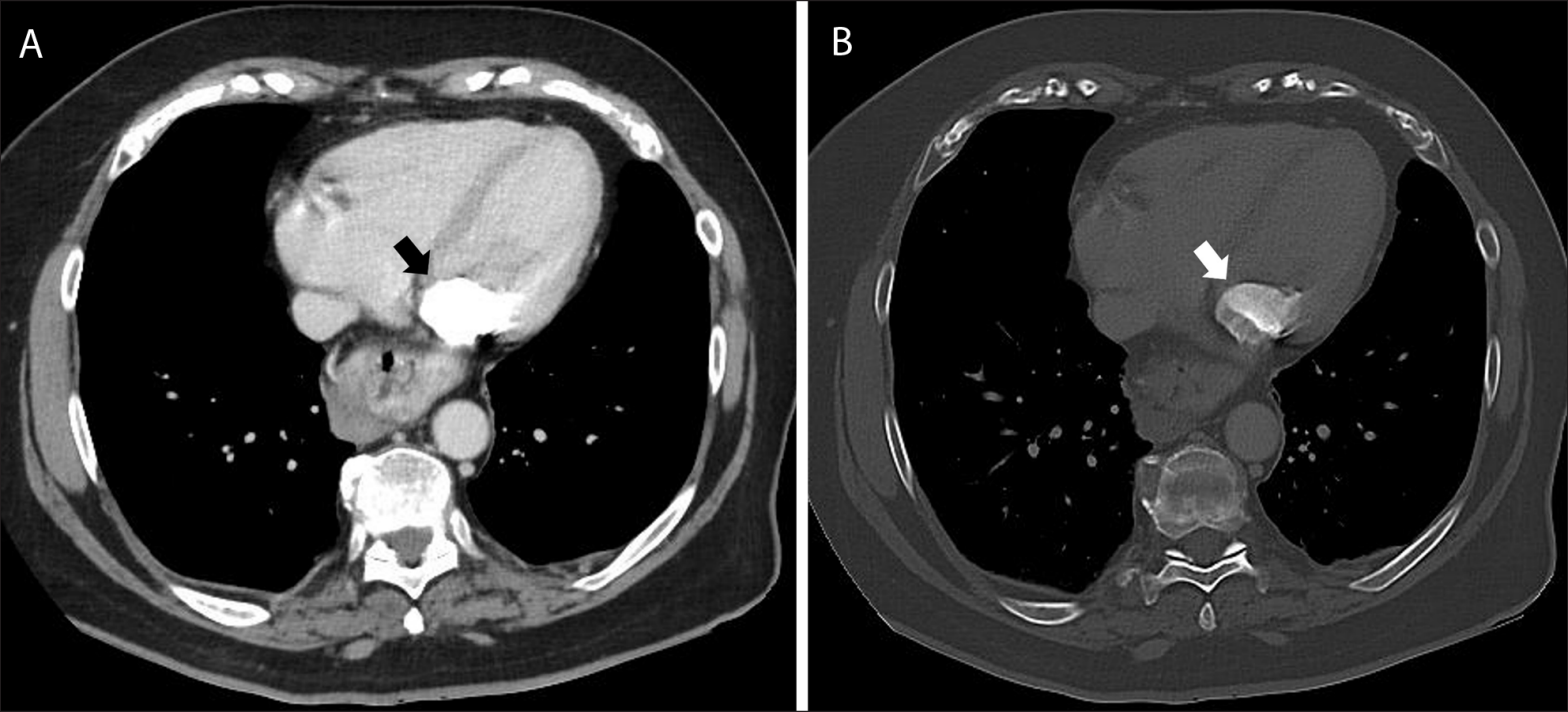


**Figure 2:**
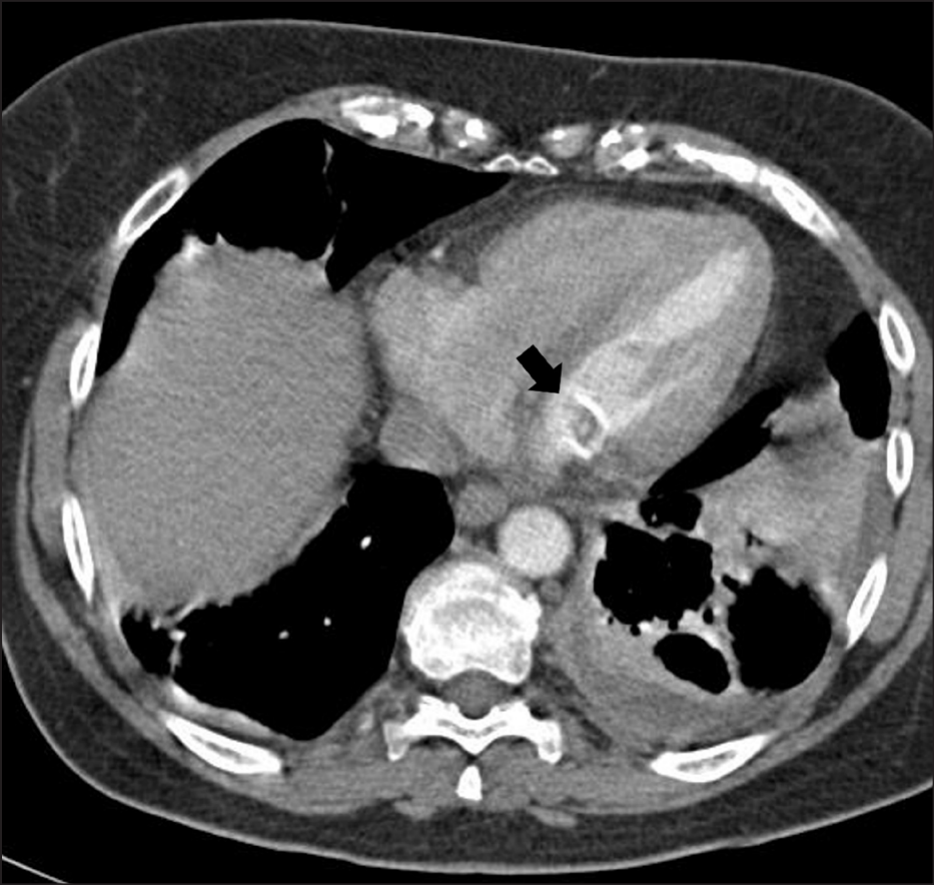


**Figure 3:**
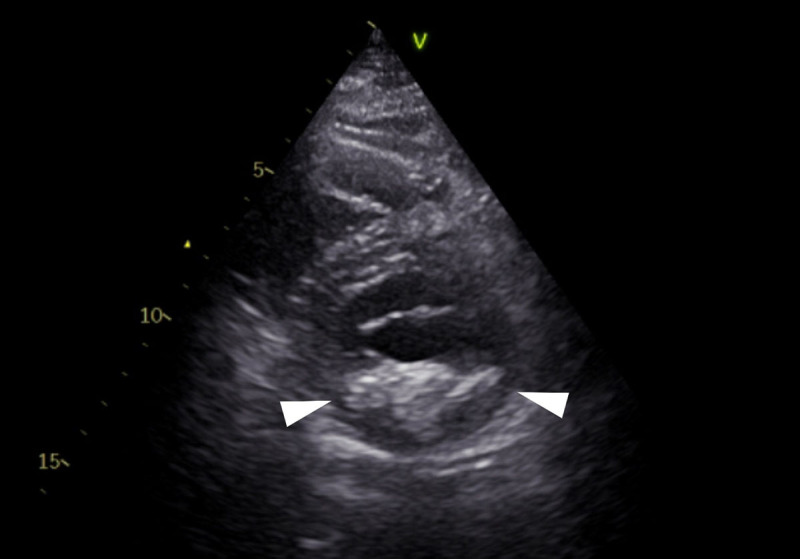


The mass was located on the posterior edge of the mitral valve, bulging into the left atrium (Figure 1A). Bone window reconstruction revealed areas of intralesional calcification (Figure 1B). A CT performed 14 years prior to the study, depicted a smaller mass with rim calcifications and a central area of low density (Figure 2). On transthoracic ultrasound, the presence of an echogenic calcified mass was confirmed (Figure 3). The lesion had smooth edges on ultrasound imaging, with no acoustic shadowing artifact. The imaging findings were in keeping with caseous calcification of the mitral valve with mitral valve insufficiency. An annual follow-up was organised and no further treatment was needed.

## Comments

Caseous calcification of the mitral annulus (CCMA) is a rare variant of mitral annular calcification (MAC) [1]. It most commonly affects elderly women, patients with a history of cardiac hypertension, chronic renal disease, or altered calcium metabolism.

CCMA mainly involves the posterior fibrous mitral annulus, where it appears as a toothpaste-like mass with a central region of caseous material and peripheral calcifications. It has a dynamic evolution, where CCMA can transform into MAC. CCMA is a benign entity, but with multimodal imaging it can be differentiated from other cardiac lesions.

Transthoracic echocardiography is considered the first-line imaging technique, allowing functional evaluation of the mitral valve. On ultrasound CCMA appears as a smooth, periannular hyperechogenic mass with a central echolucent area, consistent with a caseous content of liquefaction. It shows no acoustic shadow artifacts or vascularisation on color Doppler.

Cardiac CT has higher spatial resolution, making it most suitable for confirming calcification. On CT, CCMA will show no contrast enhancement. In case of doubt, when CT is inconsistent with a diagnosis, cardiac magnetic resonance (CMR) allows better tissue characterization. On CMR, CCMA is depicted as a mass with low-signal intensity in both T1- and T2-weighted sequences, reflecting its calcium content. Central to the mass, a zone of T1-hyperintensity can be seen. CCMA shows no contrast enhancement on first-pass perfusion sequences, unlike in tumor or abscess. However, late gadolinium-enhancement imaging may show a slight peripheral enhancement, indicating liquefactive necrosis.

CCMA is generally found incidentally, as it is usually asymptomatic. Hence, as it has a mass-like configuration, CCMA is often misdiagnosed as a cardiac tumor or abscess. Therefore, multimodal imaging is useful for diagnosis, avoiding unnecessary surgical interventions.
